# Transdiagnostic and tailored internet intervention to improve mental health among university students: Research protocol for a randomized controlled trial

**DOI:** 10.1186/s13063-024-07986-1

**Published:** 2024-03-01

**Authors:** Anne H. Berman, Naira Topooco, Petra Lindfors, Marcus Bendtsen, Philip Lindner, Olof Molander, Martin Kraepelien, Christopher Sundström, Nooshin Talebizadeh, Karin Engström, George Vlaescu, Gerhard Andersson, Claes Andersson

**Affiliations:** 1 Department of Psychology, Uppsala University, Uppsala, Sweden; 2 Centre for Psychiatry Research, Department of Clinical Neuroscience, Karolinska Institutet, & Stockholm Health Care Services, Region Stockholm, Stockholm, Sweden; 3 Department of Behavioural Sciences and Learning, Department of Biomedical and Clinical Sciences, Linköping University, Linköping, Sweden; 4 Department of Psychology, Stockholm University, Stockholm, Sweden; 5 Department of Health, Medicine and Caring Sciences, Linköping University, Linköping, Sweden; 6 Department of Global Public Health, Karolinska Institutet, Solna, Sweden; 7 Department of Criminology, Malmö University, Malmö, Sweden

**Keywords:** Depression, Anxiety, Transdiagnostic intervention, Tailored intervention, Internet intervention, University

## Abstract

**Background:**

Emerging adulthood is often associated with mental health problems. About one in three university students report symptoms of depression and anxiety that can negatively affect their developmental trajectory concerning work, intimate relationships, and health. This can interfere with academic performance, as mood and anxiety disorders are key predictors of dropout from higher education. A treatment gap exists, where a considerable proportion of students do not seek help for mood and anxiety symptoms. Offering internet interventions to students with mental health problems could reduce the treatment gap, increase mental health, and improve academic performance. A meta-analysis on internet interventions for university students showed small effects for depression and none for anxiety. Larger trials are recommended to further explore effects of guidance, transdiagnostic approaches, and individual treatment components.

**Methods:**

This study will offer 1200 university students in Sweden participation in a three-armed randomized controlled trial (RCT) evaluating a guided or unguided transdiagnostic internet intervention for mild to moderate depression and anxiety, where the waitlist control group accesses the intervention at 6-month follow-up. Students reporting suicidal ideation/behaviors will be excluded and referred to treatment within the existing healthcare system. An embedded study within the trial (SWAT) will assess at week 3 of 8 whether participants in the guided and unguided groups are at higher risk of failing to benefit from treatment. Those at risk will be randomized to an adaptive treatment strategy, or to continue the treatment as originally randomized. Primary outcomes are symptoms of depression and anxiety. Follow-ups will occur at post-treatment and at 6-, 12-, and 24-month post-randomization. Between-group outcome analyses will be reported, and qualitative interviews about treatment experiences are planned.

**Discussion:**

This study investigates the effects of a transdiagnostic internet intervention among university students in Sweden, with an adaptive treatment strategy employed during the course of treatment to minimize the risk of treatment failure. The study will contribute knowledge about longitudinal trajectories of mental health and well-being following treatment, taking into account possible gender differences in responsiveness to treatment. With time, effective internet interventions could make treatment for mental health issues more widely accessible to the student group.

## Administrative information

Note: the numbers in parentheses in this protocol refer to SPIRIT checklist item numbers; curly brackets had to be removed due to conflict with the Endnote reference system. The order of the items in has been modified to group similar items according to the journal structured protocol template.

**Table:** 

Title (1)	Transdiagnostic and tailored internet intervention to improve mental health among university students: Research protocol for a randomized controlled trial
Trial registration (2a and 2b)	2a. https://classic.clinicaltrials.gov/ct2/show/NCT05085756 2b. The trial is registered at clinicaltrials.gov. All items for the WHO TRDS can be found in that protocol, with the exception of key secondary outcomes, which are described in Table 2 in this research protocol.
Protocol version (3)	January 30, 2024
Funding (4)	The study is funded by Swedish Research Council grant number 2019–01127 to the first author.
Author details (5a)	^1^Department of Psychology, Uppsala University, Uppsala, Sweden ^2^ Centre for Psychiatry Research, Department of Clinical Neuroscience, Karolinska Institutet, & Stockholm Health Care Services, Region Stockholm, Stockholm, Sweden ^3^Department of Behavioural Sciences and Learning, Department of Biomedical and Clinical Sciences, Linköping University ^4^ Department of Psychology, Stockholm University, Stockholm, Sweden ^5^ Department of Health, Medicine and Caring Sciences, Linköping University ^6^ Department of Global Public Health, Karolinska Institutet ^7^ Department of Criminology, Malmö University, Malmö, Sweden
Name and contact information for the trial sponsor (5b)	Swedish Research Council, https://www.vr.se/english.html
Role of sponsor (5c)	The funding agency has had no role in the design of the study, nor will they have any involvement in collection, analysis and interpretation of data, and writing of manuscripts.

## Introduction

### Background and rationale (6a)

The human developmental trajectory is marked by crucial transitions from one phase to another, for example childhood to adolescence, or late adolescence to emerging adulthood [1]. For many individuals, the latter phase coincides with university studies and is characterized by exploration of identity on a personal and career level [2]. The period is also characterized by a higher rate of engagement in risky behaviors, compared to later adulthood [1]. The variability of life changes and events in emerging adulthood is associated with a high prevalence and incidence of mental health problems. About one in three university students show symptoms of depression and anxiety that can have serious negative effects on the developmental trajectory at work, in intimate relationships and for health [3, 4], and may interfere with academic performance [5]. The most prevalent disorders at this stage include mood, anxiety, and substance use, all key predictors of dropout from higher education [6]. Additional mental problems frequently co-occur with these disorders. Despite the prevalence of mental health problems, students are generally not very likely to seek help, because they either do not believe they need help, experience stigma or lack confidence that they will be helped, or find that they lack quick access to treatment [7]. Cognitive behavioral therapy (CBT) is an internationally widespread treatment method shown to be effective for common mental health disorders, particularly depression [8]. One way to lower the threshold for access to such treatment is to offer internet interventions, which have shown treatment effects for CBT that are equivalent to face-to-face treatment for diagnosis-specific depression, anxiety, and a variety of other conditions [9].

A significant overall research question is whether these internet interventions should be guided by human support. Early studies found that human guidance significantly contributed to reducing attrition as well as increasing treatment effects, and a recent review of 31 meta-analyses confirmed that just under half of reported studies showed that guidance was significantly better than treatment with no human support or minimal such support. At the same time, 9% showed that unguided interventions had better outcomes than supported ones. It is still not clear what factors indicate an obvious need for supported interventions, although the severity of mental health problems might be one indicator [10].

For the target group of university students in particular, a systematic review and meta-analysis of internet interventions to increase students’ mental health showed small between-group effects on measures of depression and no between-group differences on anxiety measures when publication bias was taken into account, where human guidance of interventions did not yield any advantage for the students [11]. In contrast, a later synthesis of data from 18 systematic reviews and meta-analyses for a wider group of adolescents and young people concluded that CBT-based interventions for depression and anxiety could be termed effective, and that involvement of human support could improve effectiveness and intervention adherence [12]. The most recent synthesis of evidence for ICBT interventions for university students, an umbrella review including seven systematic reviews or meta-analyses, found research support for expanding access to digital mental health interventions for university students, and recommended increasing rigor in future studies, including increased analysis of user data on engagement and intervention retention [13]. It thus seems that offering evidence-based guided or unguided internet interventions to students with mental health problems could potentially yield at least some improvements in mental health [3], in the best of cases, participating in such interventions could also contribute to better academic performance and quality of life during the university years, and support improved mental health even past graduation.

To summarize, over the past two decades, diagnosis-specific internet interventions have been thoroughly investigated, with moderate to large effects [9]. The evidence base for internet interventions targeting university students is cautiously positive, but currently not robust [11–13]. The overall research field on digital mental health interventions (DMHIs) has recently been moving towards experimentation with internet interventions that can address more than one condition within the same program; i.e., transdiagnostic interventions based on internet-delivered CBT (iCBT) [e.g., 14]. The advantage of such transdiagnostic interventions is that they can be offered as a “one-size fits all” treatment for individuals with the most common mental health disorders, primarily depression and anxiety. Transdiagnostic interventions for mild to moderate depression and anxiety among university students have been developed and tested in Australia, Canada, Indonesia, the Netherlands, and the UK, [14–20], and results have so far shown minor effects.

Existing research on internet interventions also yields recommendations for larger randomized controlled trials, in order to explore different effects of individual treatment components and the specific role of guidance and/or tailoring of interventions [19]. Further, studies are needed to explore the possibility that internet-delivered CBT (ICBT) for students with symptoms of mild to moderate depression or anxiety might benefit from small improvements that could be clinically relevant [21]. We do not know to what extent internet-delivered transdiagnostic interventions, guided or unguided, could reduce depression and anxiety among university students, nor how long effects might persist, but the existing evidence base suggests that ICBT interventions could have positive effects in the university student group. Additional research is needed to explore if and how transdiagnostic iCBT might yield sustainable post-treatment effects. One factor affecting the lack of positive results in earlier studies could be that treatment protocols need to be designed to deliver greater tailoring in relation to individual needs, as in precision medicine [22]. Students in higher education are clearly in need of effective mental health interventions, and it is a matter of considerable urgency to meet their needs, particularly given that students in Sweden who report mental health problems are twice as likely to drop out without completing a degree [23].

The current study builds on ongoing studies mapping mental health issues among university students in Sweden [24] within the international WHO-WMH-ICS consortium [3]. This study will recruit participants from epidemiological survey respondents at multiple universities, offering participation in a three-armed randomized controlled trial (RCT) that will evaluate guided and unguided internet-based transdiagnostic treatment for depression and anxiety, in comparison to waitlist controls. The transdiagnostic internet-delivered treatment program will also offer user-tailoring of some of the treatment content, an aspect that has shown a small, significant advantage (*d* = 0.26). in comparison to content that is tailored by clinicians [25]. An additional novel feature in this project is that it will encompass a secondary, embedded randomized “study within a trial” (SWAT) [26] that addresses the risk that some of the students in the target group will be less likely to improve in ordinary guided or unguided treatment, and may benefit from an adaptive treatment strategy, which has been shown effective in an earlier trial for insomnia among adults [27]. This embedded trial aligns with a precision medicine approach that adapts treatment content to unique individual needs for increased potential treatment benefits [22].

### Objectives (7)

The overall aim of this trial is to estimate the effects of an internet intervention for common mental health problems among university students in Sweden, who demonstrate mild to moderate symptoms of depression and/or anxiety, who are experiencing lower everyday functionality and whose treatment needs are unmet [6, 28]. Such mild to moderate mental health problems can be both syndromal (i.e., qualify for a psychiatric diagnosis) or sub-syndromal (i.e., be experienced as problematic by the individual, without qualifying for a diagnosis).

The intervention will be transdiagnostic in the sense that participants will select a main focus on depression or anxiety, where the former will entail working with two specific modules on behavioral activation, and the latter will entail working with two specific modules on anxiety exposure. Three modules, the introductory one and the concluding two, will be part of the intervention for all participants. Additionally, the intervention will be tailored in the sense that participants will select three elective modules from a menu of eight, based on their own preferences.

The study design entails offering students in the target group participation in a three-armed RCT evaluating guided or unguided versions of the intervention for a 10-week duration, in comparison to a control group who accesses the unguided version of the intervention 6 months after randomization. The embedded SWAT entails a second randomization procedure 3 weeks into the treatment, where participants identified as being at risk of failing to benefit from treatment will be randomized to receive additional guided support (in the guided arm) or standard guidance (in the unguided arm), or to continue in the guided or unguided treatment arm to which they were originally randomized. Complementary qualitative interviews are conducted to increase understanding of participants’ experiences of the specific treatment received. Students reporting suicidal ideation or behaviors are excluded from the study and referred to treatment within the healthcare system.

Primary outcomes are defined as trial arm comparisons of treatment effects on syndromal and sub-syndromal depression and anxiety. Secondary outcomes are defined as additional exploratory treatment effects related to specific emotional and behavioral changes, well-being, and treatment-related processes such as alliance and negative effects.

The specific objectives are to:

- Recruit up to 1200 eligible university students.
- Include students with mild to moderate, syndromal, and sub-syndromal symptoms of depression and/or anxiety symptoms, following brief baseline screening.
- Randomize included individuals to guided or unguided treatment, or to a waitlist control group accessing treatment 6 months after randomization.
- Collect weekly data on symptoms of depression, anxiety, and well-being during the 8-week treatment period.
- In week 3 of 8, to identify participants in the guided and unguided treatment arms, who indicate lack of improvement or worsened symptoms and to randomize them within a SWAT to an adaptive treatment strategy (ATS) or to continue as per their original randomization.
- Follow up students at post-treatment and at 6-, 12-, and 24-months post-randomization.
- Undertake qualitative evaluation of students’ experiences of the specific internet treatment received, including those randomized to unguided treatment and those randomized to the ATS.
- Statistically, we aim to compare primary outcomes between treatment arms, namely treatment effects on syndromal and sub-syndromal depression and anxiety, in relation to the primary hypothesis that both guided and unguided groups will show superior moderate-sized overall effects compared to the waitlist control group, and the secondary hypothesis assumes that the guided group will show no differences in effects in relation to the unguided group.
- Statistically, we will also compare primary outcomes for individuals in the guided and unguided treatment arms, who participated in the embedded SWAT, in relation to the hypothesis that those who received an ATS will show improved small to moderate effects in comparison to those who did not receive an ATS. A secondary hypothesis regarding the SWAT is that trial retention will be higher for individuals who received an ATS, in comparison to those did not.
- We will also conduct additional statistical evaluations, comparing secondary outcomes for the main and SWAT trials, with identical hypotheses to those formulated for the primary outcomes.

### Trial design (8)

This is a three-armed, parallel-group RCT, estimating the efficacy of a guided transdiagnostic, tailored internet treatment program for mild to moderate depression and anxiety, and of an unguided version of the same program. The efficacy of each of these programs will be estimated in comparison to a waitlisted control group. In a SWAT, participants in the guided and unguided arms of the trial who show no short-term benefit from the treatment, are included in a secondary randomization to an ATS entailing additional guidance (the guided arm) or standard guidance (the unguided arm). The study also encompasses qualitative evaluation of the treatment experience.

## Methods: participants, interventions and outcomes

### Study setting (9)

Participants will be recruited from among university students who have responded to the online baseline WHO-WMH-ICS survey [3] in Sweden in their first term of study. Recruitment to this study takes place early in the respondents’ second term of studies via a brief additional online survey of treatment eligibility. Annual follow-ups to the WHO-WMH-ICS survey take place, allowing for expanded recruitment to include invitation to survey respondents in their 4th or 6th term of study. Students are recruited at all participating universities, 15 at this writing (see https://iterapi.se/puma).

### Eligibility criteria (10)

The inclusion criteria for the main trial are as follows: self-report of mild to moderate depression and/or anxiety, operationalized as a score of at least 5 and up to and including 19 points on the 9-item Patient Health Questionnaire (PHQ-9) [29]; and/or a score of at 5 or more points on the 7-item General Anxiety Disorder questionnaire (GAD-7) [30]. The SWAT inclusion criteria are listed under SPIRIT item 11b.

The exclusion criteria for the main trial are, in the following order:

1. Current engagement in psychological treatment; and/or
2. Having initiated medication for depression and/or anxiety within the 3 weeks prior to completing the recruitment questionnaire.
3. Scores that are (a) below (too mild) or (b) above the inclusion thresholds on PHQ-9 or GAD-7 (too severe). The latter participants are asked to provide their telephone number to the study management if they would like a telephone counseling session with the PI or co-PI; or
4. Scores of 2 or 3 on the suicidal ideation item 9 in the PHQ-9; or
5. Scores on the DSM-5 CCSM short-form questionnaire [31, 32] of (a) 3 or more on the DSM-5 suicide question (domain VI, item 11), (b) and/or scores of 2 or more on one or both of the DSM-5 psychosis questions (domain VII, items 12 and 13); (c) and/or scores of 3 or more on one or both of the DSM-5 alcohol/drug questions (domain XIII, items 21 and 23).

Participants who fulfil exclusion criteria 1 or 2 do not complete the PHQ-9 and GAD-7. Participants who do not fulfill exclusion criteria 3 or 4 complete the DSM-5 CCSM short-form questionnaire for further assessment of exclusion criteria 5a-c.

### Who will take informed consent? (26a)

Informed consent will be collected electronically, via the online treatment eligibility screening survey, accessed following invitation to participate in the study. This consent form includes participation in the SWAT, as well as possible future invitations to participate in individual qualitative interviews. Consent to participate in the baseline online WHO-WMH-ICS survey has been provided earlier, including the option to consent to future linkage with higher education outcome data from the national Ladok student registry.

### Additional consent provisions for collection and use of participant data and biological specimens (26b)

Not applicable. No biological specimens are collected.

## Interventions

### Explanation for the choice of comparators (6b)

The primary comparator for the guided and unguided intervention groups is the waitlisted control group, which gains access to the unguided version of the intervention 6 months after randomization. The secondary comparator for the guided intervention group is comparison with the unguided group. The justification for this is given in the “Background and rationale (6a)” section.

### Intervention description (11a)

The intervention, titled “PUMA,” is offered to participants in all three of the trial arms and consists of eight treatment modules based on CBT, all delivered over 8 weeks via the iTerapi internet treatment platform at Linköping University [33]. Participants are recommended to work with one module per week. Modules 1, 7, and 8 are standard for all, and focus on psychoeducation, acceptance of one’s thoughts and feelings, and on strategies for self-care. The content for modules 2 and 3 depends on the participant’s choice of a depression- or anxiety-focused track and focuses on behavioral activation or exposure-based strategies, respectively. Modules 4, 5, and 6 are tailored choices from a menu of eight additional elective modules. The modules in the elective treatment menu were selected in view of evidence that university students display treatment needs related to emotion regulation, cognitive thought management, stress management, relaxation, problematic sleep patterns, social anxiety, panic anxiety, and perfectionism. Participants maintain unguided access to the program content for 2 weeks following the 8-week program. Versions of the modules used in this trial were evaluated in two earlier studies [34, 35] as well as in a more recent study evaluating self-selected tailoring as compared to clinician-selected tailoring, with a somewhat more positive outcome for the self-selected procedure [25]. For this trial, existing module content was thoroughly reviewed and revised, for adaptation to the student target group, to improve language clarity and to reduce repetition. Table 1 displays an overview of the treatment content.

**Table 1:** Intervention modules in the “PUMA” transdiagnostic, tailored ICBT program for students

Week	Module type	Theme	Title
**1a. Overview**			
1	Standard^a^	Psychoeducation	It’s about you
2	Track choice^b^	Behavioral activation I	Map your activities
2	Track choice^b^	Anxiety and GAD	Anxiety and constant worry
3	Track choice^b^	Behavioral activation II	Change through action
3	Track choice^b^	Anxiety and exposure	Anxiety
4	Tailored choice^c^	Rank #1	
5	Tailored choice^c^	Rank #2	
6	Tailored choice^c^	Rank #3	
7	Standard^a^	Acceptance	Acceptance
8	Standard^a^	Maintenance	Continue to take care of yourself
**1b. Tailored choice. All elective module options are presented to participants at the start of their treatment program. The participant then ranks the top three preferences.**			
N/A	Tailored choice^c^	Emotion and affect regulation	Everything we feel
N/A	Tailored choice^c^	Cognitive restructuring	Change your thoughts
N/A	Tailored choice^c^	Stress management	Manage stress
N/A	Tailored choice^c^	Relaxation	Relaxation
N/A	Tailored choice^c^	Sleep hygiene	Change your sleep
N/A	Tailored choice^c^	Social anxiety	Anxiety and social contact
N/A	Tailored choice^c^	Panic anxiety	Anxiety and panic
N/A	Tailored choice^c^	Perfectionism	Being perfect?

Note: Each participant’s individual treatment plan is manually coded onto the platform following randomization. For all participants, access to standard and tailored materials is given automatically, in sequence

^a^Standard: The module is provided to all participants

^b^Track choice: The module is assigned on the basis of the participant’s main problem area

^c^Tailored choice: The participant selects three modules. Access is provided manually by the guide assigned to the participant, both for the guided and unguided groups

In both the guided and unguided arms of the intervention, a guide with basic CBT skills will provide an initial telephone conversation to offer each participant an opportunity to prepare for the treatment and, optionally, to consider which of the depression or anxiety tracks to select and which three elective modules to choose. For participants in the unguided arm, the self-help nature of the intervention will be explained, and information will be given that technical questions will be answered by the treatment site support team, and that any questions about the research project should be directed to the principal investigator. Participants in the waitlist arm will be offered the self-help version of the intervention 6 months after recruitment. No initial telephone conversation will be offered waitlist participants.

Guides, hereafter also referred to as therapists, are clinical psychology students at the master’s level, or healthcare professionals with basic knowledge of CBT. Therapist guides will all be concurrent participants in a remotely delivered 11-week university-level course on psychological treatment delivered via the internet; they will deliver the treatment as part of the clinical practicum within the course. Prior to initiating treatments, therapists will complete a study-specific practical training workshop on conducting online-CBT treatment, including a study-specific treatment manual. During the treatment practicum, therapists will receive 10 weekly 90-min group supervision sessions by a licensed psychologist with experience in offering internet treatment. Throughout the treatment period, therapists will refer continually to the treatment manual and guidelines, including checklists for weekly tasks. Thereafter, therapists will provide regular written feedback on participant progress within 48 h, with an instruction to spend about 15–20 min per week per participant.

The adaptive treatment strategy offered within the SWAT will entail randomization of each participant qualifying for participation (see SPIRIT items 11b and 11c). Participants in both the guided and unguided arms will be eligible for the ATS. Participants in the guided arm will be offered additional personalized support, the nature of which is clarified in an additional telephone session with the therapist or, if the participant prefers, via written communication. The ATS for participants in the unguided arm will entail standard written guidance, as provided to participants in the guided arm of the RCT. Participants in the waitlist group will not be eligible for participation in the SWAT.

#### Procedure for inclusion in the SWAT

Participants will be included in the SWAT and randomized to the ATS when a risk of not benefitting from treatment is identified. The threshold for identifying risk will be calculated based on an algorithm that includes data from Timepoint 0 (T0; baseline trial eligibility screening), T1 (pre-treatment assessment), and T2 (mid-treatment assessment during the third week of treatment); see Table 2 for an overview of questionnaires included at each timepoint.

**Table 2:** Overview of instruments used in treatment (Tx) RCT Sweden. Inspired by Weisel et al. 2019 [36]. All followups are timed from the point of pre-Tx randomization

Nr	Phase	Category	Construct	Instrument	T0 Tx eligibility assessment	T1 Pre-Tx Assessment	Weekly during Tx	T2 Mid-Tx after 3rd Tx module	T3 End of Tx (8 w)	T4 6 m follow-up	T5 12 m follow-up	T6 24 m follow-up
1	Screen	Mental health	Demographic / mental health screen	WHO-WMH-ICS	Data from 1st term						3rd term	5th term
1.1	Information	Referral	Referral	Resources	√							
2	Screen	Tx now?	Exclusion	3 Qs	√							
2.1	Screen	Mental health	Co-morbidity	DSM-5 CCSM +	√			√	√	√	√	√
3	Screen/Weekly/ primary outcome	Mental health	Depression	PHQ-9	√	√	√	√	√	√	√	√
4	Screen/Weekly/ primary outcome	Mental health	Anxiety	GAD-7	√	√	√	√	√	√	√	√
5	Screen (as long as relevant)	Mental health	Pandemic-related anxiety	GAD-7COVID	√							
6	Screen + all phases	Wellbeing	Well-being	WHO-5	√	√	√	√	√	√	√	√
7	Pre-Tx	Tx prep	Motivational component	Telephone interview		√						
8	Pre-Tx	Tx prep	Tx expectancy	CEQ		√		√				
9	Pre-Tx	Tx prep	Attitudes towards professional help	ATSPPHS		√					√	√
10	Mid-Tx assessment	Risk factor	Risk factor Sleep	ISI		√		√	√	√	√	√
11	Mid-Tx assessment	Skill	Behavioral Activation Depression	BADS		√		√	√	√	√	√
12	Mid-Tx assessment	Skill	Managing negative thoughts	SOCT-P		√		√	√			√
13	Mid-Tx assessment	Txprocess	Tx alliance	WAI-SR for internet Tx				√	√			
14	Secondary outcome	Wellbeing	Quality of life	WHOQOLBref		√					√	√
15	Secondary outcome	Risk factor	Alcohol	AUDIT-C		√			√	√	√	√
16	Secondary outcome	Risk factor	Risk factor Self-esteem	RSES		√			√	√	√	√
17	Secondary outcome	Risk factor	Risk factor Resilience	CD-RISC-25		√			√	√	√	√
18	Secondary outcome	Risk factor	Emotion regulation	DERS-16		√			√	√	√	√
19	Secondary outcome	Risk factor	Worry	PSWQ		√			√	√	√	√
20	Secondary outcome	Risk factor	Personality	BFI-10		√					√	√
21	Secondary outcome	Health econ	Other help accessed	TIC-P		√				√	√	√
22	Secondary outcome	Txprocess	Tx satisfaction	CSQ				√ (22.1)	√	√		√
23	Secondary outcome	Txprocess	Tx negative effects	NEQ				√	√	√		√
24	Qualitative study	Txprocess	Interview guide	Qual interview						√		

Instrument abbreviations

1. *WHO-WMH-ICS survey* World Mental Health-College Student Inititative Survey

2. *DSM-5 Level 1 CCSM* Diagnostic and Statistical Manual of Mental Disorders Cross-Cutting Symptom Measure (26 items), American Psychiatric Assoc. [31, 32]

3. *PHQ-9* Patient Health Questionnaire (9 items), Kroenke et al. 2001 [29]

4. *GAD-7* General Anxiety Disorder questionnaire (7 items), Spitzer et al. 2006 [30]

5. *GAD-7- COVID* GAD-7 adapted for COVID-19 pandemic, Wahlund et al., 2020 [37]

6. *WHO-5* World Health Organization Well-being questionnaire (5 items), Topp et al. 2015 [38]

7. *Telephone interview* Research group’s original guide based on student user input (Berman, Granlund, Molander et al., 2021, unpublished)

8. *CEQ* Credibility-Expectancy Questionnaire (5 items), Devilly & Borkovec 2000 [39]

9. *ATSPPHS* Attitudes towards seeking professional psychological help scale (10 items), Surgenor, 1985 [40]

10. *ISI* Insomnia Severity Index (7 items), Morin et al., 2011 [41]

11. *BADS* Behavioral Activation for Depression Scale (25 items), Kanter et al. 2007, 2010 [42, 43]

12. *SOCT-P* Skills of Cognitive Therapy – Patient/participant version (8 items), Jarrett et al., 2011[44]

13. *WAI-SR adapted for internet* Working Alliance Inventory – Self-Report (12 items) Tracey & Kokotowitc 1989 [45]

14. *WHOQOL-Bref* World Health Organization Quality of Life Scale – Brief (26 items), Skevington et al., 2004 [46]

15. *AUDIT-C* Alcohol Use Disorders Identification Test – Consumption (3 items), Bush et al. 1998 [47]

16. *RSES* Rosenberg Self-Esteem Scale (10 items), Rosenberg, 1979; [48, 49]

17. *1CD-RISC-25* Connor-Davidson Resilience Scale (25 items), Connor-Davidson et al. 2003; Velickovic et al. 2020 [50, 51], permission granted

18. *DERS-16* Difficulties in Emotion-Regulation Scale (16 items), Swedish version Bjureberg et al., 2015 [52]

19. *PSWQ* Penn-State Worry Questionnaire (16 items), Berle et al., 2011, Swedish version, E Andersson, E Hedman et al. 2017 [53, 54]

20. *BFI-10* Big Five Inventory-10 (10 items), Rammstedt & John, 2007 [55]

21. *TIC-P* Healthcare consumption and productivity loss in patients with a psychiatric disorder (56 items); Bouwmans et al., 2013 [56]

22. *CSQ* Client Satisfaction Questionnaire (8 items), Attkisson & Zwick 1982 [57]

22.1. *CSQ, adapted* Client Satisfaction Questionnaire (8 items), adapted for mid-Tx assessment

23. *NEQ* Negative treatment effects questionnaire (20 items), Rozental et al. 2019 [58]

24. *Qualitative interview* Qualitative interview on experience of treatment intervention

The algorithm will build on data from the PHQ-9 and GAD-7 questionnaires, where data for all three timepoints will be entered for each questionnaire separately, as follows: [(T0 + T1)/2] − T2. If the outcome of the algorithm is equal to £0 for either PHQ-9 or GAD-7, or both, the participant will be randomized to the ATS, or to continue in the treatment arm to which they were assigned at the start of the main trial. See under “Interim analyses (21b)” for a more detailed description. Randomization will be conducted via SPSS version 28.0 using an outcome of the exact number of randomized individuals, stratified by group allocation to guided or unguided treatment. Participants with missing data at any of these timepoints will not be included in the SWAT since unsuccessful attempts will have already been made to encourage them to respond, and dropout from the main trial seems likely.

#### Qualitative interviews

The trial will include qualitative evaluation to obtain a deeper understanding of participants’ subjective experiences of guided, unguided, or waitlisted treatment, as well as of SWAT allocation to an ATS.

### Criteria for discontinuing or modifying allocated interventions (11b)

Lack of short-term benefit from the intervention will constitute the inclusion criterion for the SWAT. Lack of short-term benefit will be operationalized as lack of improvement or worsening of symptoms according to PHQ-9 and/or GAD-7, measured in the third week of treatment, for participants in both the guided and unguided arms. Participants eligible for SWAT participation will be included in a secondary randomization procedure. Those randomized to the ATS will receive additional human guidance if originally randomized to the guided arm, or will be given standard guidance through written secure messages, if originally randomized to the unguided arm. Participants not randomized to the ATS will continue in the original allocation to guided or unguided treatment, without any added support. Participants in the waitlist group will not be included in the SWAT.

### Strategies to improve adherence to interventions (11c)

In the guided and unguided groups, randomization to an ATS within the SWAT trial is primarily intended to minimize the risk of treatment failure. This aim includes improving intervention adherence. Additional strategies include email reminders, as well as telephone follow-ups to be initiated in the spring of 2024. As previously noted, the waitlist group will not be part of the SWAT.

### Relevant concomitant care permitted or prohibited during the trial (11d)

Throughout the trial, included participants will be free to access ordinary healthcare and/or local student mental health services as needed, i.e., seeking other healthcare resources alongside study participation is not an exclusion criterion. Within the trial, risk management and safety rules concerning suicidal ideation and/or plans have been established. In the first step, the study team will receive an automatic notification from the study platform if a participant endorses increased suicidal ideation compared to the baseline measure (i.e., a score of 2 or higher on the suicidal ideation item on item 9 of the PHQ-9, which will be administered at all assessment points). In the second step, a protocol to assess risk, provide referral resources, and discuss safety planning has been established for the study, and will be applied as follows for participants in both treatment groups: If a participant scores either a 2 or a 3 on the PHQ-9 suicide item, the treatment therapist (1) will send a message inside the study platform to assess the participant’s well-being and level of risk; (2) will communicate that the participant may receive a phone call from the therapist; (3) if the participant does not respond to the message so that risk can be assessed, the therapist will contact the participant via telephone to discuss the current situation and possible needs for referral, (4) if the therapist deems it warranted, they will contact the participant immediately by phone. Steps 1–4 will also be implemented in cases where the therapist assesses a higher risk for the participant based on communication in the platform, not conditional on raised suicidal ideation. For all steps, the principal investigator will be available to discuss severity level assessment, safety planning, and referral options on a case-by-case basis. A list of available healthcare resources in the community (e.g., student services, suicide prevention hotline, crisis text line, primary care provider) will be provided by the study as needed.

### Provisions for post-trial care (30)

Standard care is available within the national healthcare system in Sweden.

### Outcomes (12)

#### Primary outcome

The trial has two co-primary outcomes, assessed in all participants regardless of treatment received. The primary contrasts are guided vs. waiting list and unguided vs. waiting list.

- Anxiety, which will be measured using GAD-7 (total score).
- Depression, which will be measured using PHQ-9 (total score).

Although participants choose a primary treatment focus on anxiety or depression, most are expected to exhibit baseline symptoms for both disorders. For this reason, the two co-primary outcomes are assessed and analyzed among all participants.

Primary outcomes are assessed 10 weeks post-randomization (T3 post-treatment measure), 6 months post-randomization (T4), 12 months post-randomization (T5), and 24 months post-randomization (T6). After T4, participants in the waiting list control group will be offered the unguided version of the interventions; thus, we will use T3 and T4 measures for contrasts among all three groups and all follow-up interval measures for contrasts between guided and unguided groups (adjusted for baseline measures).

**Fig. 1:**
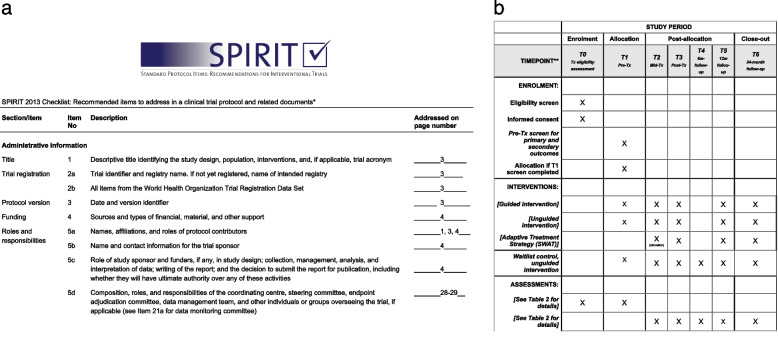
SPIRIT checklist (a) and schedule of enrolment, interventions, and assessments (b)

**Fig. 2:**
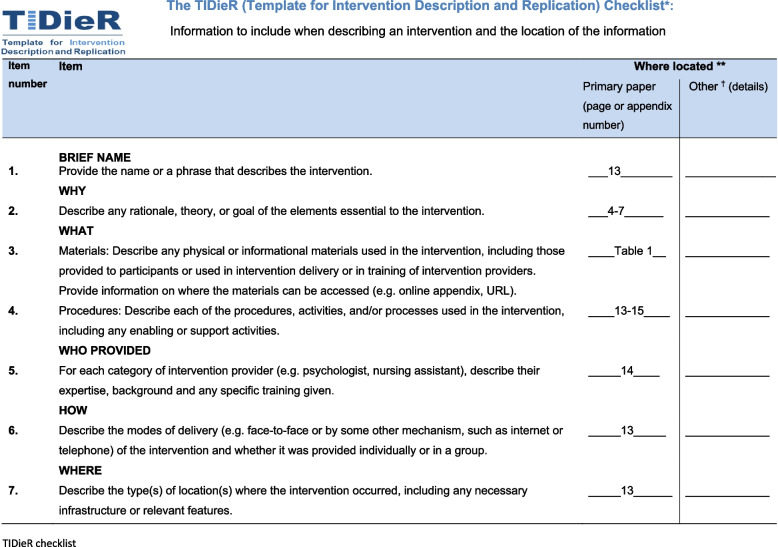
TIDieR checklist for intervention description and replication

**Fig. 3:**
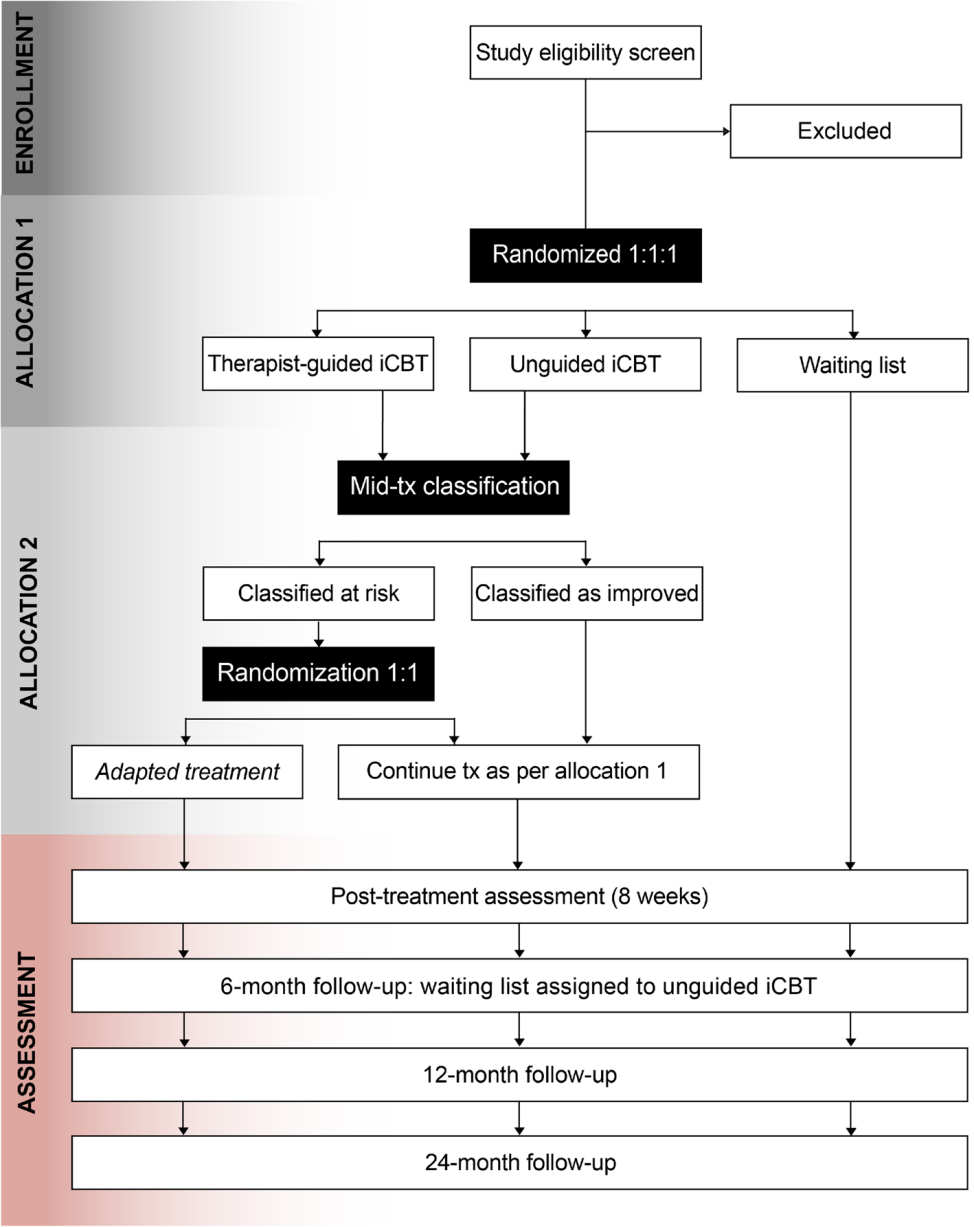
CONSORT RCT flowchart

See Fig. 1a for a complete SPIRIT checklist, Fig. 1b for the participant timeline, Fig. 2 for a TIDieR checklist regarding the intervention description, Fig. 3 for a CONSORT flow diagram, Table 1 for an overview of the intervention modules, and Table 2 for a complete, referenced list of the assessment instrument used in the study.

#### Secondary outcomes

The trial also has nine secondary outcomes, assessed in all participants regardless of treatment received. Following the same rationale as for the primary outcomes contrasts among all three groups will be done using measures at T3 and T4, while T5 and T6 measures will be used to contrast guided and unguided groups (adjusted for baseline measures). All secondary outcomes are listed in Table 2 in order of chronological administration to participants.

- Externally and behaviorally oriented outcomes
  - Alcohol consumption, which will be measured using AUDIT-C (T3-T6).
  - Comorbidity, which will be measured using DSM-5 CCSM (T3-T6).
  - Resilience, which will be measured using CD-RISC-25 (T3-T6).
  - Sleep, which will be measured using ISI (T3-T6).
  - Well-being, which will be measured using WHO-5 (T3-T6).
- Internally oriented outcomes
  - Emotional regulation, which will be measured using DERS-16 (T3-T6).
  - Personality, which will be measured using BFI-10 (T3-T6).
  - Self-esteem, which will be measured using RSES (T3-T6).
  - Worry, which will be measured using PSWQ (T3-T6).
- Mediator outcomes
  - Behavioral activation-depression, measured using BADS (T2-T3).
  - Behavioral activation-depression, measured using BADS (T2-T3).

### Participant timeline (13)

See Fig. 1 for participants’ timeline through the trial including allocation to the SWAT. Qualitative interviews will be conducted starting midway through the RCT and are estimated to take about 30–60 min per interview.

### Sample size (14)

We calculated the required sample size based on the smallest effect size on primary outcomes that we did not want to miss, with respect to the contrast between the guided intervention and waiting list control at 6 months post-randomization (T4). We estimated this effect size to 0.3 Cohen’s *d*. With a significance level of 0.05 (alpha) and power of 0.8 (beta), a two-sided *t*-test would require 176 individuals per group, giving a total of 528. Assuming an attrition rate of 40% at T4, this means that we should recruit at least 880 participants. Notwithstanding this power calculation, we hope to extend our recruitment sample to up to 1200 participants if possible, given additional possible attrition after T4 and the importance of reporting outcomes over a longer term than 6 months.

Regarding analysis of the SWAT comparing adapted versus continued standard treatment for at-risk participants, earlier work evaluating for insomnia [33] suggests that between-group differences in final scores corresponding to *d* = 0.4 are possible, which would require *n* = 100 + 100 at-risk participants, i.e., well within the recruitment target.

### Recruitment (15)

Potential participants will be recruited from among all respondents to the WHO-WMH-ICS mental health survey, currently administered to first-term students in multi-year educational programs at 15 universities and colleges in Sweden. Additional higher education institutions may be added. The invitation to survey respondents is sent out one term later, 4 to 6 months after completion of the survey, a time period sufficient for mental health status to change. This is the rationale for inviting *all* respondents rather than restricting invitations to individuals who already exhibit symptoms of mild to moderate depression and/or anxiety in the baseline epidemiological survey. Up to three reminders are sent after invitation.

## Assignment of interventions: allocation

Consenting participants are included in the study if their symptoms of depression and/or anxiety, as reported in the T0 treatment eligibility screening questionnaire (see Table 2), correspond to a mild to moderate level of severity. If they complete the pre-treatment assessment at T1 (see Fig. 1 and Table 2), they are allocated to one of the three study arms.

### Sequence generation (16a)

A randomization sequence is generated in blocks of 12 (3 × 4) and then matched to the list of participants who have qualified for randomization.

### Concealment mechanism (16b)

The randomization sequence is generated via an R script (programmed by PhL), which ensures a priori allocation concealment.

### Implementation (16c)

Author PhL, who is part of the research team but does not participate in weekly meetings and is not involved in everyday survey or trial implementation, conducts the randomization and communicates the list to author NT.

## Assignment of interventions: blinding

### Who will be blinded (17a)

Participants, guides, and authors AHB and NT are not blinded to trial arm allocation. However, the researcher responsible for generation of the randomization sequence is blinded to the actual allocation of participants. Authors who will conduct analyses (MB, PL, and OM) are all blinded to trial arm allocation.

### Procedure for unblinding if needed (17b)

Since blinding does not hold for those directly involved in everyday implementation and participation in the trial, no unblinding procedure has been conceived.

## Data collection and management

### Plans for assessment and collection of outcomes (18a)

Data are collected at treatment eligibility screening, pre-treatment, mid-treatment, post-treatment, and 6, 12, and 24 months after randomization. In addition, weekly measures of depression, anxiety, and well-being are collected during treatment (see Table 2).

### Plans to promote participant retention and complete follow-up (18b)

There is a concern that participant attrition from follow-up will be higher than expected. Research interns, available from the spring of 2024, will be tasked with contacting participants at follow-up times, pending ethical approval.

### Data management (19)

All data are collected online and stored on a secure server at Uppsala University, according to a pre-registered data management plan (DMP). During trial implementation, all data collection from T1 onwards, including weekly measures, are stored on the iTerapi platform at Linköping University [33]. Once all data collection is completed, all trial data will be exported for analysis at Uppsala University. Authors NT and AHB monitor missing data on an ongoing basis.

### Confidentiality (27)

Data are stored in accordance with the DMP for the project and are managed according to the General Data Protection Regulation (GDPR) that covers personal data management in the European Union. Raw trial data are not currently shared with the WHO-WMH-ICS consortium.

## Analysis

### Statistical methods for primary and secondary outcomes (20a)

We will keep participants in the groups to which they were randomized in all analyses (intention-to-treat). We will conduct both available data analyses and analyses with missing data imputed using multiple imputations with chained equations (generating 200 datasets using 30 iterations of predictive mean matching). Both available data and imputed data analyses will be used to interpret findings.

All models of primary and secondary outcomes will be estimated using both Bayesian inference and maximum likelihood estimation [59]. The former will estimate the posterior distribution over effect estimates, while the latter will be used for null hypothesis testing. We will use (half) Student’s *t* priors for covariates, fixed intercepts, and error terms (centered at 0, with a scale of 2.5 and 3 degrees of freedom). Adaptive intercepts in multilevel models will be given standard normal priors. For point estimates, we will report the median of the posterior distribution of each parameter of interest (i.e., group contrasts), along with 95% compatibility intervals defined by the 2.5 and 97.5% percentiles of the posterior distributions. We will use the posterior probability of effect and *P*-values for our scientific inference.

The two co-primary outcomes, anxiety and depression, will be standardized and modeled using a multilevel linear regression model. The models will include a time-by-group interaction with time modeled as a dummy categorical variable (0 = T3, 1 = T4, 2 = T5, 3 = T6), as will group (0 = waiting list, 1 = unguided, 2 = guided). The models will be adjusted for baseline (T1) measures of the outcome variables (respectively) using a baseline-by-time interaction term and further adjusted for age, gender, and a binary variable indicating HBTQ (0 = No, 1 = Yes). Adaptive intercepts will be added to the models at the participant and university level. The linear combination of coefficient estimates for the group and time-by-group covariates will be used as effect estimates (i.e., differences between groups at the different follow-up intervals). For our primary contrasts (guided vs. waiting list and unguided vs. waiting list), we will report effects at T3 and T4. For our exploratory contrast (guided vs. unguided), we will report effects at all four follow-up intervals.

The secondary outcomes will be modeled similarly to the primary outcomes. The only exception is comorbidity, measured using DSM-5 CCSM, which results in the classification of participants concerning several conditions. We will use multilevel negative binomial regression to analyze the number of conditions participants are positive for (and fall back to Poisson regression if the outcome is not over-dispersed). In addition, DSM-5 CCSM is not measured at baseline (T1) but is measured at trial eligibility assessment (T0); thus models for DSM-5 CCSM will be adjusted for T0 measures.

### Interim analyses (21b)

The inclusion procedure for the SWAT can be defined as an interim analysis. Participants in their third week of treatment will be requested to respond to the questionnaires listed under column T2, Table 2. At the end of the third week, responses to the PHQ-9 and the GAD-7 are extracted and compared to baseline (T0) and T1 responses on these questionnaires. The algorithm for calculation of lack of benefit is conducted separately for the PHQ-9 and the GAD-7, such that scores on at least one of these measures that are equal to or less than 0, regardless of the participant’s chosen treatment track, lead to SWAT randomization ([PHQ-9_TO_ + PHQ-9_T1_/2] − PHQ-9 _T2_ ≤ 0) and/or ([GAD-7_TO_ + (GAD-7_T1_)/2] − GAD-7 _T2_ ≤ 0). This procedure means that the inclusion procedure for the SWAT is broad and that individuals who have improved in one type of symptom but have not improved, or worsened, in the other, can be randomized to an ATS. No other interim analyses will be conducted at this timepoint.

Regarding stopping rules, no such rules have been stipulated in advance. However, we will monitor suicide thoughts weekly via item 9 in the PHQ-9. Participants who respond with a score of ≥ 2 will be contacted by telephone for a dialog about any need for additional help. The rationale for this procedure is that external events (e.g., failing an exam) can trigger a higher score on this item, and it can thereafter decline in the next weekly measure. If a need arises for a participant to seek additional help, we will refer them and will discuss whether or not they will continue in the trial. See under “Relevant concomitant care permitted or prohibited during the trial (11d)” for further details concerning safety procedures and relevant concomitant care during the trial.

### Methods for additional analyses (e.g., subgroup analyses) (20b)

#### Mediator models

We will estimate the mediated effects of the guided and unguided interventions (vs. waiting list) with respect to two potentially mediating factors (behavioral activation-depression and managing negative thoughts). We will use a causal inference framework, using Bayesian inference to estimate the natural direct effect and natural indirect effect (as per the definitions of Pearl [60]). We will report on the posterior distributions of these two effects. For each primary outcome (anxiety and depression), we will estimate mediation models for each mediator separately, as well as one model with both mediators. Mediator measures will be standardized and modeled using linear regression, using the same adjustments as in the primary outcome models (i.e., outcomes measured at baseline, age, gender, and a binary variable indicating HBTQ). The mediation models will estimate both the mediated effects from T2 mediators to T3 outcomes, and T3 mediators to T4 outcomes.

#### Ancillary analyses

We will conduct effect modification analyses by introducing interaction covariates into the regression models with respect to age, gender, HBTQ, and each respective outcome at baseline. The posterior probability of interaction effects and the Watanabe-Akaike information criterion (WAIC) will be used for scientific inference from the effect modification models.

Systematic attrition to follow-up at the different time intervals will be studied by estimating the odds ratio of not responding conditional on age, gender, HBQT, and the two co-primary outcome variables measured at baseline.

#### SWAT analyses

To study the effects of the adaptive treatment strategy (ATS), meaning that participants who do not improve by the third week in treatment are randomized to extra support, based on primary outcomes, we will estimate the same multilevel models as in the primary outcome analyses but include only participants randomized to guided or unguided trial arms, for whom extra support was indicated. We will replace the time-by-group contrast in the multilevel models with a time-by-SWAT contrast and add an adjustment for main contrast group (guided vs. unguided). Exploratory analyses will add an additional interaction with main contrast group (time-by-group-by-SWAT) to estimate the differential effect of extra support conditional on guided or unguided mode of intervention delivery.

#### Exploratory analyses

This trial will generate additional data not analyzed in the primary analyses. These data will be used in exploratory analyses with the aim of producing evidence for future refinements of interventions and intervention delivery. These exploratory analyses include:

- A process evaluation of the interventions, including participants’ experiences of support, treatment expectancies, satisfaction and alliance, and any negative treatment effects. Specific treatment process outcomes for all RCT participants include treatment expectancy (CEQ), attitudes towards professional help (ATSPPHS), treatment alliance (WAI-SR for Internet treatment), treatment satisfaction (CSQ), and negative effects of treatment (NEQ); see Table 2.
- Treatment process is also measured in terms of completed treatment modules and % of completed skills practices. Trial process outcomes for all RCT participants concern adherence.
- To the assessment plan, measured in terms of % completed measures at post-treatment; SWAT adherence measures include % of participants who are randomized to receive additional therapist support for the remainder of the treatment period (weeks 4 to 8). For participants who have given their consent in the preceding WHO-WMH-ICS survey, secondary outcomes will also include annual data on students’ academic records via LADOK, the Swedish national documentation system of higher education outcomes, specifically academic success, changes in academic study focus, or interruption of studies. Qualitative interviews concerning experience of treatment outcome and treatment-seeking pathways will complement secondary outcome measurement.
- Additional trial process-related outcomes include overall treatment interest and uptake, measured in terms of the percentage (%) of participants who respond to the study invitation (interest) as well as participate in treatment (uptake).
- An analysis of primary outcomes using all measures from all assessments during the intervention period, and follow-up data, with time modeled as numeric rather than categorical as in the primary analyses (with and without quadratic term for time).

### Methods in analysis to handle protocol non-adherence and any statistical methods to handle missing data (20c)

We will conduct both available data analyses and analyses with missing data imputed using multiple imputations with chained equations (generating 200 datasets using 30 iterations of predictive mean matching). Both available data and imputed data analyses will be used to interpret findings.

### Plans to give access to the full protocol, participant-level data and statistical code (31c)

The full protocol is contained in the approved ethical application (in Swedish) and is available in the supplementary material. Requests for participant-level data should be directed to the principal investigator (PI), Professor Berman, anne.h.berman@psyk.uu.se.

## Oversight and monitoring

### Composition of the coordinating center and trial steering committee (5d)

The trial steering committee meets weekly via encrypted Zoom. Additional research team meetings take place ad hoc every 1–3 months, and in-person meetings are held at least once a year for strategic planning.

### Composition of the data monitoring committee, its role and reporting structure (21a)

Monitoring concerning any adverse events, clinical or data-related, occurs weekly at the core research team meetings. Additional data monitoring meetings occur monthly with author NoT, who is operationally responsible for data management together with author AHB. Data planning meetings occur yearly or ad hoc with the Uppsala University Data Protection Officer, at the Legal Affairs Division, Office for Information Provision, Registry and University Archives.

### Adverse event reporting and harms (22)

Respondents who have been excluded from the RCT due to mental health issues too severe based on baseline scoring over the inclusion thresholds, are offered a telephone counseling session with principal investigator AHB, a licensed clinical psychologist and psychotherapist, or with co-principal investigator CA, a social worker with extensive clinical experience, with the aim of identifying appropriate referral to treatment at student mental health centers or within the ordinary healthcare system.

Adverse events and harms during the RCT are measured with the Negative Experiences Questionnaire (NEQ), [61], and trial safety procedures include monitoring of symptom levels. Suicidal ideation is addressed according to the treatment protocol, formulated by authors NT, AHB, and GA. In brief, this protocol requires that guides conduct a weekly check on the level of depressive and anxiety symptoms. Additionally, suicidal ideation scores of ≥2 on item 9 of the PHQ-9 are flagged in the platform system and the treatment coordinator (NT) sends a message instructing the therapist how to proceed to contact the participant according to manual guidelines to assess the participant’s specific ideation content and risk of forming a concrete suicide plan. The PI (AHB) is available for consultation, e.g., if the total PHQ-9 score is significantly higher compared to earlier weeks, and/or if the guide has had a conversation with the participant that requires a supervision session beyond ordinary, weekly group supervision.

### Frequency and plans for auditing trial conduct (23)

Trial conduct is actively monitored via the core research team’s weekly meetings. Our management structure for this trial is slim, and the Project Management Group meets once every week. This group also manages trial steering as well as data monitoring and ethics. In this trial, we have two primary endpoint measures of outcome, PHQ-9 and GAD-7. We considered GCP monitoring for this trial but opted out due to their requirement that we use one endpoint measure as a primary outcome.

### Plans for communicating important protocol amendments to relevant parties (e.g., trial participants, ethical committees) (25)

Changes to the protocol require that the PI address proposed amendments to the Swedish National Ethical Review Authority https://etikprovningsmyndigheten.se/en/. See item 24.

### Dissemination plans (31a)

Publication and conference presentation plans are regularly drawn up and revised as needed at the core research team’s weekly meetings. Trial results will be disseminated via publications, conferences, participating universities’ media platforms, and the project website, https://www.psyk.uu.se/Divisions/clinical-psychology/national-assessment-and-e-health-interventions-for-mental-health-problems-among-university-students/.

## Discussion

Reducing the treatment gap for Swedish university students is a matter of high priority, particularly since Swedish students have worse mental health problems compared to their peers not attending university [62]. This project conducts research on internet interventions and expands the reach of mental health treatment for students, since more participants can be treated per treatment provider than via in-person treatment. With time, dissemination of internet interventions into ordinary care via student mental health clinics can make such interventions for mental health issues far more widely accessible to the student group.

Two specific innovations in this study are worthy of note. Firstly, the internet intervention program we are evaluating is a transdiagnostic, tailored program focused on treatment of depression and/or generalized anxiety disorder (e.g., [36]), with some comorbidity. This is important because comorbidity is highly prevalent among university and college students and impairments in daily role tasks, associated with common mental health problems in this population, are more severe the greater the extent of comorbidity [63]. Secondly, this project will provide knowledge about what might be possible in Sweden to push mental health development forward, to improve societal health and well-being on a larger scale in the short- and long-term. The knowledge gained from the clinical trial described in this protocol will include primary outcome data from the main RCT, long-term follow-up of the guided and unguided groups, and analysis of the adapted treatment strategy evaluated in the SWAT, in addition to treatment and trial process data such as intervention adherence. We hope that our findings will contribute to expanded future access to treatment for university students, supporting student health clinics in meeting the challenges of providing evidence-based interventions for common mental health problems, for an ever-growing number of students. The qualitative studies will contribute to additional knowledge about student experiences of internet treatment.

An additional future benefit of this study is the generation of data that can be used to develop a precision treatment modeling method that can contribute to developing personally adapted treatment configurations. The predictors available will consist of a rich set of self-report variables found in prior studies to be predictors of response to CBT; e.g., socio-demographic factors; history, triggers, and symptom of mental disorders; comorbid disorders/symptoms; stress and adversity; and personality traits/temperament [64]. Such a model could potentially facilitate automated referral of larger numbers of university students with common mental health problems to a tailored, precision treatment that will increase the chances of their recovery, and enhance completion of studies, building of relationships and self-care.

### Clinical significance

The high potential for alleviating suffering from mental health problems during emerging adulthood, among university students, has been emphasized throughout the protocol. We hope that the intervention content can be transferable to an appropriate platform following conclusion of the trial, pending positive outcomes. We will continue to monitor any adverse effects of the intervention and continue to implement routines for clinical action in case of decline in participant stability while participating in the internet interventions, including waitlist control group participants.

## Trial status

Recruitment for a pilot study, reported separately, was completed on October 19, 2021. Recruitment for the main trial began in mid-March 2022 and will continue until statistical power is estimated to have been achieved. The current protocol is version 3, dated January 30, 2024.

## Data Availability

Data may be available to collaborators on request to the PI, Professor Berman, anne.h.berman@psyk.uu.se.

## Abbreviations

ATS: Adaptive treatment strategy
CBT: Cognitive behavioral therapy
DMP: Data management plan
GAD-7: General Anxiety Disorders-7 questionnaire
GDPR: General Data Protection Regulation
ICBT: Internet-based CBT
PHQ-9: Patient Health Questionnaire-9
PI: Principal Investigator
RCT: Randomized controlled trial
SWAT: Study within a trial
WHO-WMH-ICS: World Health Organization, World Mental Health International College Student Initiative
